# The Thirty Gigahertz Instrument Receiver for the QUIJOTE Experiment: Preliminary Polarization Measurements and Systematic-Error Analysis

**DOI:** 10.3390/s150819124

**Published:** 2015-08-05

**Authors:** Francisco J. Casas, David Ortiz, Enrique Villa, Juan L. Cano, Jaime Cagigas, Ana R. Pérez, Beatriz Aja, J. Vicente Terán, Luisa de la Fuente, Eduardo Artal, Roger Hoyland, Ricardo Génova-Santos

**Affiliations:** 1 Instituto de Física de Cantabria (IFCA), Avda. Los Castros s/n, Santander 39005, Spain; E-Mail: ortizgd@ifca.unican.es; 2 Departamento Ingeniería de Comunicaciones (DICOM), Universidad de Cantabria, Plaza de la Ciencia s/n, Santander 39005, Spain; E-Mails: villae@unican.es (E.V.); juanluis.cano@unican.es (J.L.C.); jaime.cagigas@erzia.com (J.C.); ana.perez@erzia.com (A.R.P.); beatriz.aja@unican.es (B.A.); josevicente.teran@unican.es (J.V.T.); luisa.delafuente@unican.es (L.F.); artale@unican.es (E.A.); 3 Instituto de Astrofísica de Canarias (IAC), Vía Láctea s/n, La Laguna 38205, Spain; E-Mails: rjh@iac.es (R.H.); rgs@iac.es (R.G.-S.)

**Keywords:** instrumentation, astronomy, polarization, cosmic microwave background, systematic errors

## Abstract

This paper presents preliminary polarization measurements and systematic-error characterization of the Thirty Gigahertz Instrument receiver developed for the QUIJOTE experiment. The instrument has been designed to measure the polarization of Cosmic Microwave Background radiation from the sky, obtaining the Q, U, and I Stokes parameters of the incoming signal simultaneously. Two kinds of linearly polarized input signals have been used as excitations in the polarimeter measurement tests in the laboratory; these show consistent results in terms of the Stokes parameters obtained. A measurement-based systematic-error characterization technique has been used in order to determine the possible sources of instrumental errors and to assist in the polarimeter calibration process.

## 1. Introduction

In 1964 Penzias and Wilson measured by chance a noise-like signal [1] that turned out to be the Cosmic Microwave Background (CMB). This radiation is the remaining footprint of the Big Bang and was postulated by Gamow, Alpher, and Herman in the late 1940s [2]. Ultra-sensitive radio astronomy instruments have been used since then to characterize the CMB. Space missions like COBE [3] in the late 1980s, WMAP [4] in the early 2000s and more recently, the PLANCK mission [5,6], have been dedicated to the analysis of temperature and polarization anisotropies of the CMB. Moreover, ground-based experiments, such as QUIET [7] and BICEP [8,9], have been developed to measure the CMB polarization to increasingly higher sensitivity with the aim of measuring the B-mode polarization pattern predicted by inflationary models of the early Universe. 

The present study is focused in the Q-U-I Joint Tenerife (QUIJOTE) experiment. This ground-based experiment has been designed to measure the polarization of the CMB and other galactic and extragalactic signals at medium and large angular scales in the frequency range from 10 to 47 GHz [10,11]. The project consists of two telescopes and three instruments: the Multi-Frequency Instrument (MFI), operating at 10–20 GHz, the Thirty-GHz Instrument (TGI) and the Forty-GHz Instrument (FGI). These are able to obtain the polarization state of the incoming electromagnetic radiation [12] by measuring the Q, U, and I Stokes parameters. Preliminary polarization measurements performed in the laboratory and a simplified method of systematic-error [13] characterization to assist in the calibration [14] of the QUIJOTE TGI polarimeter is presented in this paper. Two polarimeter units have been measured to obtain the detected output voltages, which depend on the polarization of the incoming signal. Furthermore, the measurements of the polarimeter response have been used to characterize the systematic errors of each polarimeter by using a system-level parametrical model. The reported polarization measurements and systematic-error analysis is a preliminary laboratory task with the general goal of learning about the measured signals and instrumental errors that will be present when the complete instrument is installed in its cryostat first and on the telescope for the commissioning stage. The behavior of the receivers will change owing to the cryogenic cooling of their Front-Ends, the telescope action, and by the use of real excitation signals coming for the sky, but the reported measurement and error analysis methods will possibly also be applied in those conditions.

This document is divided into five sections. The first is an introduction followed by a description and analysis of the TGI polarimeter in Section 2. Section 3 focuses on the phase-adjustment and polarization measurement methodology, and provides representative examples. A simplified systematic-error characterization technique is presented and discussed in Section 4, and, finally, Section 5 draws general conclusions.

## 2. Tgi Quijote Polarimeter

The ground-based QUIJOTE experiment is currently being operated at Teide Observatory (2400 m, a.s.l, Canary Islands, Spain) with a multi-frequency instrument (MFI) characterizing the CMB within the frequency range 10–20 GHz. Two other instruments are under development: the Thirty-GHz Instrument (TGI), and the Forty-GHz Instrument (FGI), working in the 26–36 GHz and 35–47 GHz frequency bands respectively [15]. These experiments will undertake two surveys: a wide survey covering 20,000 deg^2^ reaching a sensitivity of ~15 µK/beam with the MFI, and a deeper survey of 3000 deg^2^ with a sensitivity of ~4 µK/beam with the MFI and better than 1 µK/beam with the TGI and the FGI. As QUIJOTE is a multi-frequency experiment, the beam size depends on frequency (in the particular case of TGI the beam size is 0.36°), but in the previous values of sensitivity are referred to a 1 solid degree beam size. These data will probe multipoles roughly between multipoles l = 10 and l = 200 (survey of 3000 square deg. with an angular resolution of 1°) providing essential information about the polarization of low-frequency galactic foregrounds, and about the amplitude of the cosmological B-mode signal. 

### 2.1. TGI Polarimeter Theoretical Behavior

**Figure 1:**
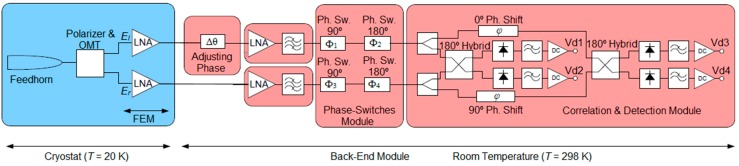
TGI polarimeter block diagram.

The TGI polarimeter block diagram for one pixel is shown in Figure 1, which shows a cold stage module inside a cryostat (20 K) and a Back-End module at room temperature (298 K). The cryogenic part is made up of a feed-horn, a polarizer, an orthomode transducer (OMT), and two Low-Noise Amplifiers (LNAs) in the Front-End Module (FEM). Outside the cryostat, operating at room temperature, the Back-End Module (BEM) is composed of an Phase adjusting (PA) component, two Gain and Filtering Modules, the Phase Switch Module (PSM), and the Correlation and Detection Module (CDM). 

In this study, the calculation of the Stokes parameters is achieved from the detected voltage waveforms of each independent detector. These waveforms are determined by the successive phase-states provided by the phase-switching module. The polarizer, together with the OMT, provides left-hand and right-hand circular polarization output signals, which, properly combined and detected, enable us to obtain the parameters. The PSMs alternate the phase differences between branches between 0/180º and 0/90° to generate four independent phase states. These modules minimize the leakage among the Stokes parameters and overcome the 1/f noise and different systematic errors in the receiver. Using a Cartesian coordinate system, the Stokes parameters are defined by:

(1) $$I={\left|E_{X}\right|}^{2}+{\left|E_{Y}\right|}^{2}$$

(2) $$Q={\left|E_{X}\right|}^{2}-{\left|E_{Y}\right|}^{2}$$

(3) $$U=2\cdot ℜ\left({E_{X}}^{*}\cdot E_{Y}\right)$$

(4) $$V=2\cdot ℑ\left({E_{X}}^{*}\cdot E_{Y}\right)$$

where *E_X_* and *E_Y_* are the orthogonal electrical field components in the coordinate system received by the feed-horn. The parameter *V* is assumed to be zero since the CMB is considered not to be circularly polarized [16] and is not measured. Therefore, assuming the incoming signal at the receiver to be linearly polarized , a translation to a circularly polarized wave is performed with the square quad-ridge waveguide polarizer [15] combined with the OMT, which splits the left- and right-hand circular components to accomplish the behavior of a septum polarizer [17] with the benefit of avoiding its usual bandwidth limitation.

The combination of the 90°- and 180°-phase switches in Figure 1 (*Φ_1_*, *Φ_3_* and *Φ_2_*, *Φ_4_* respectively) provides four phase states in each branch resulting in sixteen phase states in the overall pixel. As different combinations in the module cause redundant states, the pixel behavior is easily analyzed in terms of the phase difference between the two branches of the pixel *Φ_T_*, given by:

(5) $$\Phi_{T}=\Phi_{B_{2}}-\Phi_{B_{1}}=\left(\Phi_{3}+\Phi_{4}\right)-\left(\Phi_{1}+\Phi_{2}\right)$$

where *Φ_B2_* and *Φ_B1_* are the insertion phases of the lower and upper branches of the PSM shown in Figure 1. The input signals to the PSM, *E_l_* and *E_r_*, are the outputs of the OMT [17] and are defined by:

(6) $$E_{l}\propto \frac{1}{\sqrt{2}}\cdot \left(E_{X}+j\cdot E_{Y}\right)$$

(7) $$E_{r}\propto \frac{1}{\sqrt{2}}\cdot \left(E_{X}-j\cdot E_{Y}\right)$$

Assuming ideal behavior of the scheme in Figure 1 and the previous equations, the detected voltages for the different phase states as a function of the Stokes parameters are easily obtained (see Table 1, where K is a proportionality constant related to the gain of the polarimeter). 

**Table 1:** Detected voltages for an *x*-axis linearly polarized signal.

Φ_T_	V_d1_	V_d2_	V_d3_	V_d4_
0º	K·(I + Q)	K·(I − Q)	K·(I + U)	K·(I − U)
90º	K·(I + U)	K·(I − U)	K·(I − Q)	K·(I + Q)
180º	K·(I − Q)	K·(I + Q)	K·(I − U)	K·(I + U)
270º	K·(I − U)	K·(I + U)	K·(I + Q)	K·(I − Q)

Alternatively the detected voltages can be expressed as a function of *Φ_T_* by means of the following equations:

(8) $$V_{d1}=K\cdot \left(I+Q\cdot \cos\Phi_{T}+U\cdot \sin\Phi_{T}\right)=K\cdot \left(I+I_{p}\cdot \cos\left(\Phi_{T}+\tan^{-1}\left(U/Q\right)\right)\right)$$

(9) $$V_{d2}=K\cdot \left(I-Q\cdot \cos\Phi_{T}-U\cdot \sin\Phi_{T}\right)=K\cdot \left(I+I_{p}\cdot \cos\left(\Phi_{T}+\tan^{-1}\left(-U/-Q\right)\right)\right)$$

(10) $$V_{d3}=K\cdot \left(I+U\cdot \cos\Phi_{T}-Q\cdot \sin\Phi_{T}\right)=K\cdot \left(I+I_{p}\cdot \cos\left(\Phi_{T}+\tan^{-1}\left(-Q/U\right)\right)\right)$$

(11) $$V_{d4}=K\cdot \left(I-U\cdot \cos\Phi_{T}+Q\cdot \sin\Phi_{T}\right)=K\cdot \left(I+I_{p}\cdot \cos\left(\Phi_{T}+\tan^{-1}\left(Q/-U\right)\right)\right)$$

where *I_p_* is given by:

(12) $$I_{p}=\sqrt{U^{2}+Q^{2}}$$

## 3. Preliminary Polarization Measurements

A broad-band linearly polarized input signal was used to excite two pixels of the QUIJOTE TGI. The detected voltages at the BEM outputs were recorded to calculate Stokes parameters according to the polarization of the input signal. The receiver chains are two of the pixels that will be installed in the TGI at Teide Observatory. The FEM cryogenic LNAs have not been included in the receivers owing to the excess of signal power at room temperature. In any case, the functionality of the polarimeters is not affected by this since the optomechanics (feed-horn, polarizer, and OMT), PSMs, and CDMs are the more relevant modules in the overall polarimeter functionality. 

### 3.1. Measurement Test-Bench

**Figure 2:**
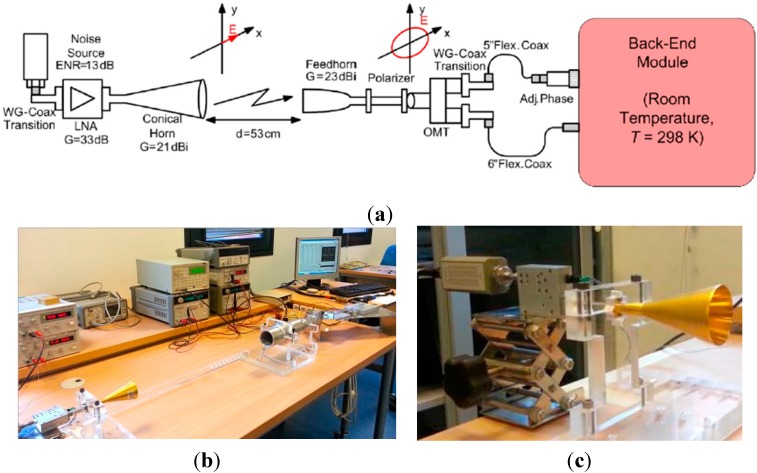
TGI Polarimeter measurement test bench. (**a**) Receiver functionality test bench (BEM details in Figure 1). (**b**) Polarimeter test bench with *x*-axis linearly polarized source. (**c**) *y*-axis polarized source.

The polarimeter phase-adjustment and measurement process is carried out by exciting the receiver with broadband x-axis and y-axis linearly polarized signals respectively. Figure 2 shows a sketch (a) and pictures of the measurement test-bench (b), and the noise source (c) used as excitation sources in the laboratory. 

The broadband noise-like linearly polarized signal is accomplished using a 346C (option K01) noise source model from Agilent Technologies (Santa Rosa, CA, USA) together with a conical horn having a rectangular waveguide input. The orientation of the rectangular waveguide port determines the orientation of the radiated E-field within the cross-polarization limits of the horn. Between the noise source and the horn, a broadband LNA raises the signal power while the distance between the transmitting antenna and the receiver feed-horn is adjusted to avoid compression in the detectors. The two signals split up in the OMT outputs and are correlated in the last module of the chain. Since both signals are affected by a phase imbalance due to small differences in the electrical paths, one of the branches of the receiver is provided with a commercial PA coaxial component, which enables the minimization of the phase difference between branches. The detected voltages, once amplified with video amplifiers in a differential configuration, provide output levels lower than 10 V. These signals are sampled by a 24-bit resolution Data Acquisition System (DAS) [18] (PXI-4495 from National Instruments, Austin, TX, USA).

As has been explained in the previous section, the combination of the 90°- and 180°-phase switches provides four phase states in each branch, resulting in sixteen redundant phase states in the overall pixel. The phase shift between the two branches of the pixel (*Φ_T_*) can be provided in a sequence given by the repetition of the four fundamental values (0°, 90°, 180°, and 270°) until covering the sixteen possible phase-states. The detected voltages of the polarimeter have been measured, covering all the phase switch states, in order to calculate the Stokes parameters after processing the obtained values. An acquisition period of one second has been used to cover the complete sequence of sixteen phase states. 

### 3.2. Measurement and Phase-Adjustment Process

The polarization measurement method is a real-time process in which the detected signals are acquired by the DAS in the successive 16 phase states provided by the PSM. The resulting detected signals can be analyzed by means of a Fast Fourier Transform (FFT) in order to obtain their amplitude and phase and also to calculate the Stokes parameters. The measured waveforms are sinusoids but, as the detected voltages provide only four points per cycle, they are represented as saw-tooth waveforms in this work. To illustrate this situation and how the 16 phase states are achieved, Table 2 shows the values of one of the four detected voltages (v_d_) measured in the resulting sequence of phase-states. 

If the FFT of v_d_ is called V_d_, a simplified version of v_d_ can be reconstructed by means of the DC component (V_d_[0]) and the fundamental harmonic (V_d_[4]) following Equation (13):

(13) $${\text{v}}_{\text{d}i\text{\_FFT}}={\text{V}}_{\text{d}i}\left[\text{0}\right]+\text{abs}\left({\text{V}}_{\text{d}i}\left[\text{4}\right]\right)\text{*cos}\left(2\pi ft+{\text{tan}}^{\text{-1}}\left(ℑ\left({\text{V}}_{\text{d}i}\left[\text{4}\right]\right)/\left(ℜ\left({\text{V}}_{\text{d}i}\left[\text{4}\right]\right)\right)\right)\right)$$

f = 4 Hz in this case because in one second the sequence of four phase shift fundamental values between branches (0º, 90º 180º, and 270º) is repeated four times. For the example in Table 2, these values are V_d_[0] = 2.59 V and V_d_[4] = 2.395 − 0.054i V respectively. The rest of the FFT harmonics are not taken into account because they can be considered as residual terms. By comparison of Equations (8)–(12) with Equation (13), it is easy to identify the Stokes parameters [19] and some figures of merit related to them as the *Q*/*U* isolation (*ISO*) and the polarization percentage (*Pol_perc*) of the signal. Other parameters, such as the *phase* of the signal, can also be calculated in order to get the phase-error of the polarimeter that is directly related with *ISO*. Equations (14)–(19) define all these parameters. 

**Table 2:** Detected voltage values for each of the phase-states forming a saw-tooth waveform.

State	*Φ_T_* (deg)	*Φ_B2_* (deg)	*Φ_B1_* (deg)	v_d_ (V)
0	0	0	0	5.28
1	90	90	0	2.71
2	180	180	0	0.19
3	270	270	0	2.54
4	0	90	90	4.66
5	90	180	90	2.68
6	180	270	90	0.17
7	270	0	90	2.54
8	0	180	180	5.26
9	90	270	180	2.62
10	180	0	180	0.18
11	270	90	180	2.47
12	0	270	270	4.67
13	90	0	270	2.63
14	180	90	270	0.17
15	270	180	270	2.66

(14) $$Q_{i}=\frac{ℜ\left(V_{d1}\left[4\right]\right)}{V_{d1}\left[0\right]}\text{, }\frac{\text{-}ℜ\left(V_{d2}\left[4\right]\right)}{V_{d2}\left[0\right]}\text{, }\frac{-ℑ\left(V_{d3}\left[4\right]\right)}{V_{d3}\left[0\right]}\text{, }\frac{ℑ\left(V_{d4}\left[4\right]\right)}{V_{d4}\left[0\right]}$$

(15) $$U_{i}=\frac{ℑ\left(V_{d1}\left[4\right]\right)}{V_{d1}\left[0\right]}\text{, }\frac{-ℑ\left(V_{d2}\left[4\right]\right)}{V_{d2}\left[0\right]}\text{, }\frac{ℜ\left(V_{d3}\left[4\right]\right)}{V_{d3}\left[0\right]}\text{, }\frac{\text{-}ℜ\left(V_{d4}\left[4\right]\right)}{V_{d4}\left[0\right]}$$

(16) $$I_{i}=\;\sqrt{U_{i}^{2}+Q_{i}^{2}}$$

(17) ISOi = 10*log10(abs(UiQi)) (dB)

(18) Phasei=(180π)*atan(ℑ(Vdi[4])ℜ(Vdi[4])) (Deg)

(19) $$Pol\_perc_{i}\;=\text{ 100*}I_{i}\text{ (\%)}$$

**Figure 3:**
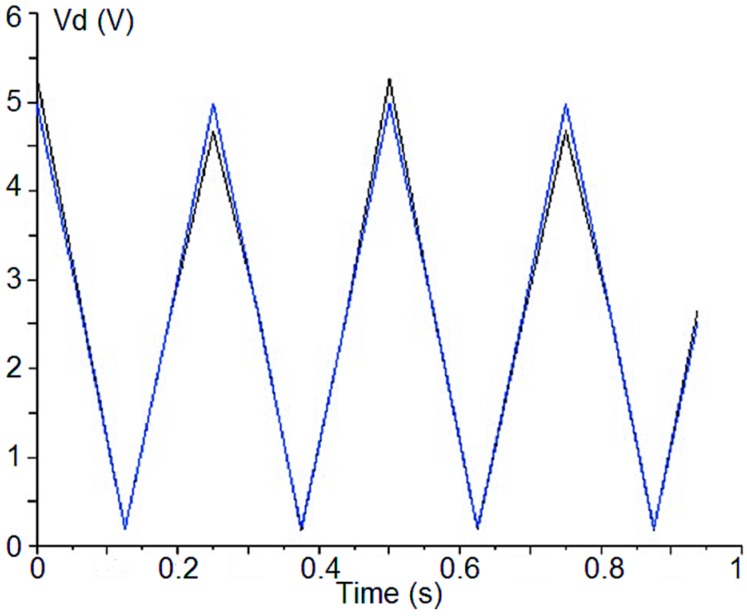
Detected voltage waveform from Table 2 values (black trace) and the resulting waveform calculated from the FFT of the measured values (blue trace).

As can be observed in Equation (19), the *I_i_* value represents the polarization percentage as the parameters are normalized by the total intensity that is given by V_di_[0] (see Equations (8)–(12) and Equation (13)). The index *i* goes from 1 to 4, representing each detector output of the polarimeter. Figure 3 shows the measured waveform of the detected voltage in Table 2 (black trace) and the resulting waveform calculated from the FFT main harmonic values (blue trace). 

**Figure 4:**
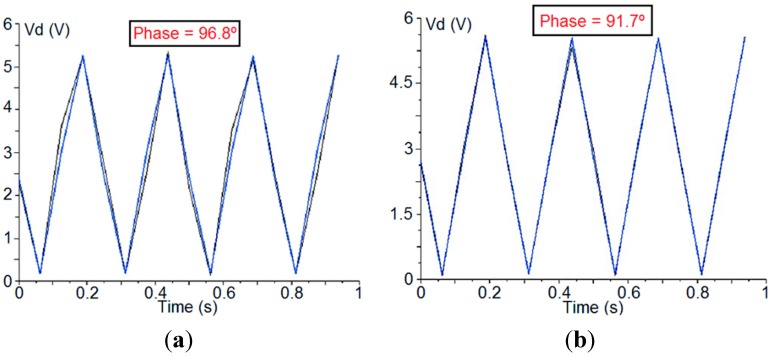
Detected signal before (**a**) and after (**b**) phase-error correction.

The PA component setting is to minimize the phase-error over the detected signals. This error comes from both the electrical paths of the two polarimeter branches, including the PSM, and also the CDM. These errors are difficult to avoid because they come mainly from tolerances in the fabrication of every component of the polarimeter. In order to illustrate the effect of the adjusting phase over the waveform of a detected signal, Figure 4 shows one detected signal before and after applying the phase correction. In the reported case, the phase of the signal should be 90°, so the phase error has decreased from 6.8° to 1.7°.

The PA component has a different effect on each detected voltage, so the following practical examples show how to measure polarization when instrumental errors are present. 

### 3.3. Practical Measurement Examples

**Figure 5:**
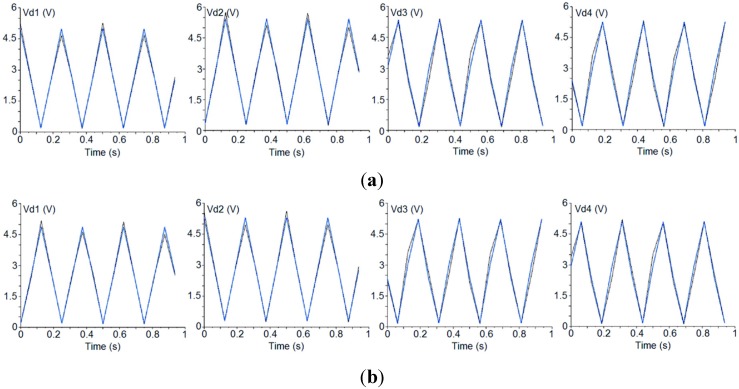
First polarimeter example: Detected signals resulting from the phase-adjustment process with an *x*-axis polarized input signal (**a**) and measurement process with a *y*-axis polarized input signal (**b**).

A first measurement example is illustrated in Figure 5 where the upper part (a) shows the detected signals after the phase-adjustment process, exciting the polarimeter with a 100% x-axis polarized signal (Figure 2b), and the lower part (b) shows the detected signals when using a 100% y-axis polarized signal (Figure 2c) and the previously achieved phase adjustment. Table 3 shows the parameters (Equations (14)–(19)) achieved from the detected voltages shown in Figure 5. In the left columns *E_Y_* = 0, so it should be measured that *I* = *Q* and *U* = 0. In the right columns *E_X_* = 0, so we should get *I* = −*Q* and *U* = 0. 

**Table 3:** Parameters calculated from the detected signals shown in Figure 5 (Phase in Deg. and ISO in dB).

Detector	U_x_	Q_x_	Phase_x_	ISO_x_	I_x_	U_y_	Q_y_	Phase_y_	ISO_y_	I_y_
1	−2.1·10^−^^2^	0.92	−1.295	−16.46	0.92	2.2·10^−2^	−0.93	178.64	−16.25	0.93
2	−1.0·10^−^^2^	0.89	179.34	−19.41	0.89	1.2·10^−2^	−0.89	−0.79	−18.61	0.89
3	0.13	0.93	−82.19	−8.63	0.94	−0.13	−0.93	97.79	−8.64	0.94
4	0.11	0.94	96.78	−9.21	0.94	−0.11	−0.94	−83.18	−9.22	0.95

It can be seen that the PA component provides correct results in detectors 1 and 2 (good isolation and phase-error values), whereas detectors 3 and 4 remain uncorrected. In fact, it has been seen that, by using this component, it is possible to adjust the phase of only one pair of detectors (the one corresponding to V_d1_ and V_d2_ or V_d3_ and V_d4_) leaving the other pair uncorrected. This behavior arises because the polarimeter presents two main sources of phase error (the electrical paths of the polarimeter branches, including the PSM, that affects the four detectors and the 90°-phase-shifter of the CDM that affects only detectors 3 and 4) and one correction factor (the adjusting-phase component) that is able to correct only the error coming from the electrical paths of the polarimeter branches. So, in the general case, one pair of detectors will be corrected while the other pair will remain affected by the error in the 90º-phase-shifter of the CDM. Therefore, the need for redundant measurements using four detectors per polarimeter enables the following measurement solution to be proposed.

Until now, the 16 states provided by the PSM have been used, but the choice of only four independent states with the lowest phase-error is now considered. In principle there are 256 combinations of four independent states that could be analyzed. An exhaustive search of the optimal combination can be implemented in the FPGA that is used to perform the measurements but, for simplicity, here we have selected the last four phase states of the sequence (states numbered from 12 to 15 in Table 2). They provide less error than others due to the lower difference between the detected values when *Φ_T_* is 90 and 270°, as can be observed in Table 2 by comparing states 13 and 15 with the pairs 1–3, 5–7 and 9–11. Table 4 shows the parameters measured using these four independent states and a 100% *x*-axis polarized excitation signal (left columns) and a 100% *y*-axis polarized excitation signal (right columns). By comparing these results with those of Table 3, the improvement achieved in the results is clear, thus providing more accurate measurement of the incoming polarization by using the four detectors of each polarimeter. 

**Table 4:** Parameters calculated by using only the states numbered from 12 to 15 in Table 2 (Phase in Deg. and ISO in dB).

Detector	U_x_	Q_x_	Phase_x_	ISO_x_	I_x_	U_y_	Q_y_	Phase_y_	ISO_y_	I_y_
1	5.72·10^−^^3^	0.89	0.37	−21.91	0.89	−1.24·10^−3^	−0.9	−179.92	−28.62	0.9
2	1.07·10^−^^2^	0.87	−179.29	−19.09	0.87	−1.62·10^−2^	−0.86	1.08	−17.26	0.86
3	1.13·10^−^^2^	0.94	−89.31	−19.22	0.94	−2.87·10^−3^	−0.97	90.17	−25.27	0.97
4	−1.22·10^−^^2^	0.98	89.27	−18.95	0.98	−6.6·10^−3^	−0.95	−90.41	−21.48	0.95

**Figure 6:**
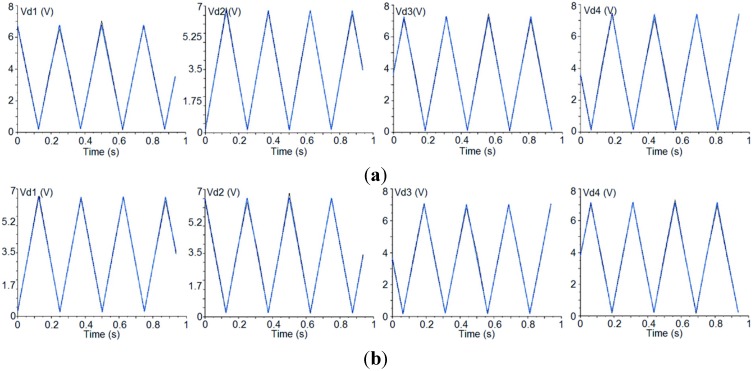
Second polarimeter example: Detected signals resulting from the phase-adjustment (**a**) and measurement (**b**) processes.

On the other hand, some of the TGI polarimeters show a low enough phase-error to be used with the complete sequence of phase states. An example of these polarimeters with low phase-error is shown in Figure 6, where the upper part (a) shows the detected signals after the phase-adjustment process, exciting the polarimeter with a 100% *x*-axis polarized signal, and the lower part (b) shows the detected signals when using a 100% *y*-axis polarized signal and the previously achieved phase adjustment. 

The parameters calculated from the signals of Figure 6 are presented in Table 5. In this case the detector presenting the highest phase error is the number 4 but it is considered low enough to measure polarization, as the polarimeter still has to be calibrated to correct the remaining systematic errors.

**Table 5:** Parameters calculated from the detected signals shown in Figure 6 (Phase in Deg. and ISO in dB).

Detector	U_x_	Q_x_	Phase_x_	ISO_x_	I_x_	U_y_	Q_y_	Phase_y_	ISO_y_	I_y_
1	3.71·10^−3^	0.94	0.23	−23.9	0.94	6.73·10^−3^	−0.92	179.58	−21.33	0.92
2	4.61·10^−3^	0.95	−179.73	−23.25	0.95	4.43·10^−2^	−0.93	−0.27	−23.19	0.93
3	−1.08·10^−3^	0.96	−90.07	−29.18	0.96	8.84·10^−3^	−0.94	89.47	−20.35	0.94
4	2.8·10^−2^	0.96	91.67	−15.35	0.96	−2·10^−2^	−0.94	−88.87	−16.68	0.94

In the previous measurements it is possible to see a polarization percentage different from 100% (*I* parameters should be equal to 1) when the excitation signal is 100% polarized and the instrumental offsets have been previously corrected. An external error source as one small part of unpolarized signal due to the free-space length between the noise source horn and the polarimeter horn (see Figure 2b) could be the reason for that. In the following we will consider 3% of the input signal to be unpolarized. In such a case, we should have measured the same offset level in the four detectors but the detected signals shows different offset values. The reason for that will be considered in the following section.

## 4. Systematic Errors Characterization

In this section, a simplified method for the polarimeter systematic-error characterization is presented. The method is based on the implementation of a polarimeter system-level parametric model in an electrical simulator and the optimization of such parameter values in order to fit the previous measurement results. The parameters represent the main amplitude and phase errors that can appear in some parts of the polarimeter, mainly owing to fabrication tolerances. In this work, ADS software from Agilent Technologies has been used to implement, simulate, and optimize the model parameters. A calibration method has been developed in parallel to this work by the QUIJOTE collaboration to cancel the systematic errors coming from the PSM. The method is based on fitting the error of each detected signal by means of the analysis of four equivalent phase states, derived from all sixteen. As the method presented here is more related to static systematic errors, both techniques may be considered as complementary. 

### 4.1. Polarimeter Parametric Model

**Figure 7:**
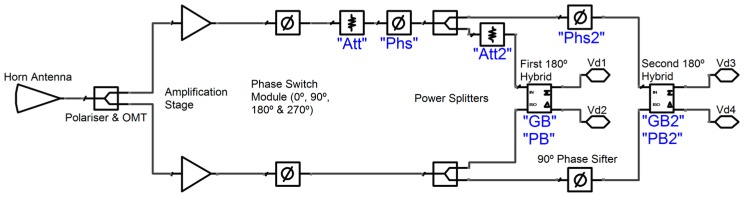
Simplified diagram of the polarimeter parametric model.

A simplified diagram of the system-level parametric model is shown in Figure 7. Eight parameters have been added (marked blue in Figure 7) to the system-level model in order to fit the imperfections achieved in the polarimeter measurements. 

Two parameters ‘Att’ and ‘Att2’ have been added to characterize the gain errors of the polarimeter. These errors can be produced by gain differences of the amplification stages (“Att”) but also by the poor isolation of the wave-guide power splitters (“Att2”), as these kinds of circuits have an ideal isolation between outputs of 6 dB. The branches of the 90-deg Phase-Shifter in the CDM do not present a gain-error parameter because this element is designed to have good matching and low insertion loss. Another two parameters, “Phs” and “Phs2”, characterize the phase errors in the polarimeter branches provided by the PSM and the 90-deg Phase-Shifter. Four more parameters, “GB”, “PB”, “GB2”, and “PB2”, characterize the gain- and phase-imbalance errors in the first and the second 180º hybrids. The reported model does not take into account either nonlinear effects or external error sources such as the unpolarized part of the input signal previously cited. The non-linearities are avoided by inserting a low enough input power to the polarimeter model, whereas the external error sources are modeled by adding a 3% of unpolarized input noise to the detected signals.

When the aforementioned eight parameters are equal to zero, the parametric model is assumed to be ideal. In order to test this assumption, Table 6 shows the model simulation results using four different linearly polarized excitation signals with polarization angles of 0° (*E_Y_* = 0, *I* = *Q*, *U* = 0), 45° (*E_Y_* = *E_X_*, *I* = *U*, *Q* = 0), 90° (*E_X_* = 0, *I* = −*Q*, *U* = 0), and 135° (*E_Y_* = −*E_X_*, *I* = −*U*, *Q* = 0). The parameters defined by Equation17 (*ISO* in dB), 18 (*Phase* in Degrees), and 19 (*Pol_Perc* in %) corresponding to each detector and excitation signal are reported in Table 6. It is rather obvious that the achieved values represent an ideal case that could not be found in real measurements. 

**Table 6:** Ideal parametric model simulation results using four excitation signals with polarization angles of 0°, 45°, 90°, and 135°.

**Pol. Angle**	**_1_**	**ISO_1_**	**Phase_1_**	**Pol_Perc_2_**	**ISO_2_**	**Phase_2_**
0°	100.00	−60.7	5·10^−^^5^	100.00	−60.6	−180
45°	100.00	−58.4	−90	100.00	−58.4	90
90°	100.00	−60.7	−180	100.00	−60.7	5·10^−5^
135°	100.00	−65.8	90	100.00	−65.8	−90
**Pol. Angle**	**Pol_Perc_3_**	**ISO_3_**	**Phase_3_**	**Pol_Perc_4_**	**ISO_4_**	**Phase_4_**
0°	100.00	−122.2	−90	100.00	−122.3	90
45°	100.00	−62.2	−180	100.00	−62.2	3·10^−5^
90°	100.00	−121.4	90	100.00	−122.4	−90
135°	100.00	−62.2	−3·10^−^^5^	100.00	−62.2	180

### 4.2. Simulation Examples Based on Measurement Results

**Figure 8:**
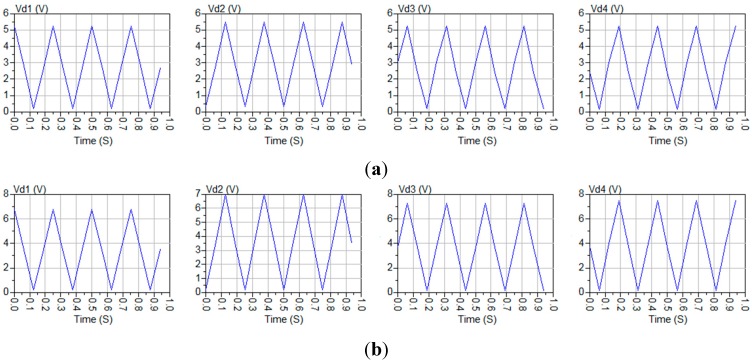
Optimized parametric model simulation results. (**a**) Fit of the situation in Figure 5a; (**b**) Fit of the situation in Figure 6a.

Two previous measurement examples have been used to illustrate the technique: The first measurement example is that reported in Figure 5a, where a polarimeter case is presented in which a horizontally polarized excitation signal gave rise to a much higher phase-error for the detectors 3 and 4 than for the detectors 1 and 2. The second example is that reported in Figure 6a, which is another polarimeter case in which the phase error is much lower than in the previous one when the excitation is a horizontally polarized excitation signal. The parametric model has been optimized to fit the *Phase* and *Pol_Perc* values of the detected signals in both cases. The values achieved by the optimized model present an almost negligible error (lower than 0.001%) when compared with that of Table 3 and Table 5 (left). Figure 8 shows the detected signals achieved from the optimization of the parametric model in both situations. 

The model fits both situations very well by means of the model parameters of Table 7. 

**Table 7:** Model parameters fitting the measurement results in Figure 5a.

Parameter	Value (Figure 8a)	Value (Figure 8b)	
Att (dB)	1.80	0.75	
Att2 (dB)	1.00	0.90	
GB (dB)	−0.54	0.29	
GB2 (dB)	−0.19	0.32	
Phs (°)	0.98	−0.25	
Phs2 (°)	−8.27	−0.55	
PB (°)	−0.37	−0.07	
PB2 (°)	−0.49	0.89	

The optimization process is not very time-consuming because the offset of the detected signals (*Pol_Perc*) is mainly determined by the four gain-error-related parameters (“Att”, “Att2”, “GB”, and “GB2”), while the phase is mainly determined by the four phase-error-related parameters (“Phs”, “Phs2”, “PB”, and “PB2”).

An examination of the parameter values in Table 7 would seem to show that the offsets are produced mainly by the gain difference between the branches of the polarimeter (parameters “Att” and “Att2”), while the phase errors of detectors 3 and 4 in Figure 8a can be attributed to the 90° Phase Shifter (the parameter “Phs2” shows an error of –8.3°) which cannot be corrected by using the PA component. On the other hand, as can be also observed in Table 7, the phase-errors are much lower (<1°) in Figure 8b than in the previous case, thus providing much higher isolation values (see Table 5), while gain-errors lower than 1 dB can be attributed to the poor isolation of the wave-guide power splitters. 

## 5. Conclusions

Preliminary polarization measurements in the laboratory and a simplified systematic-error analysis methodology for the QUIJOTE TGI have been described. The TGI polarimeters can obtain the Stokes parameters of an incoming wide-band noise-like linearly polarized signal by means of a real-time measurement and phase-adjustment method. Each polarimeter provides four detected waveforms allowing the independent calculation of the Stokes parameters simultaneously. Two different measurement examples, one showing a higher instrumental error than the other, have been reported to show how it is possible to achieve *Q*/*U* isolation values of around −20 dB, equivalent to a phase error lower than 0.5° which is considered to be small enough to assure the quality of scientific data. A simplified systematic-error characterization technique, based on the implementation of a polarimeter parametric model, has been presented. The method reported here provides information about the possible error sources, in both amplitude and phase. The gain errors can be attributed to poor isolation conditions, whereas the phase errors are mainly due to tolerances in the fabrication of every component of the polarimeter. This methodology focuses on static systematic errors, so it can be considered as complementary to calibration techniques more related to dynamic errors coming from the PSM.
